# In the tripartite combination *Botrytis cinerea*–Arabidopsis–*Eurydema oleracea*, the fungal pathogen alters the plant–insect interaction via jasmonic acid signalling activation and inducible plant-emitted volatiles

**DOI:** 10.1007/s10265-021-01273-9

**Published:** 2021-03-18

**Authors:** Luisa Ederli, Gianandrea Salerno, Mara Quaglia

**Affiliations:** Department of Agricultural, Food and Environmental Sciences, University of Perugia, Borgo XX Giugno, Perugia, 06121 Italy

**Keywords:** Arabidopsis, *Botrytis cinerea*, *Eurydema oleracea*, Jasmonate signalling pathways, Plant‐emitted volatiles

## Abstract

In ecosystems, plants are continuously challenged by combined stress conditions more than by a single biotic or abiotic factor. Consequently, in recent years studies on plant relationships with multiple stresses have aroused increasing interest. Here, the impact of inoculation with fungal pathogens with different lifestyles on Arabidopsis plants response to the following infestation with the invasive crop pest *Eurydema oleracea* was investigated. In particular, as fungal pathogens the necrotroph *Botrytis cinerea* and the biotroph *Golovinomyces orontii* were used. Plants exposed to *B. cinerea*, but not to *G. orontii*, showed reduced herbivore feeding damage. This difference was associated to different hormonal pathways triggered by the pathogens: *G. orontii* only induced the salicylate-mediated pathway, while *B. cinerea* stimulated also the jasmonate-dependent signalling, which persisted for a long time providing a long-term defence to further herbivore attack. In particular, the lower susceptibility of *B. cinerea*-infected Arabidopsis plants to *E. oleracea* was related to the stimulation of the JA-induced pathway on the production of plant volatile compounds, since treatment with VOCs emitted by *B. cinerea* inoculated plants inhibited both insect plant choice and feeding damage. These results indicate that necrotrophic plant pathogenic fungi modulate host volatile emission, thus affecting plant response to subsequent insect, thereby increasing the knowledge on tripartite plant–microbe–insect interactions in nature.

## Introduction

In nature, plants continuously interact with other biotic factors including pathogenic fungi and herbivorous insects and many studies have been carried out considering each plant–other organism interaction as a separate and independent event. However, this approach is simplistic and does not consider the more realistic contexts in which plants are simultaneously exposed to several concurrent stresses. In the latter case, plants response is the result of a complex process in which the attack by an organism induces a genetic reprogramming that alters plant interactions with the other biotic components.

In this context, infections by pathogenic fungi have shown both positive and negative impacts on the plant immunity to herbivorous insects, by changing the nutritional characteristics of the host and thus influencing insect feeding behaviour. Thus, infection with the fungus *Sclerotium rolfsii* Sacc. showed to increase soluble sugars and lower total phenol content in the peanut leaves, resulting in increased survival and growth of the herbivore *Spodoptera exigua* (Hübner) (Lepidoptera: Noctuidae) (Cardoza et al. 2003). On the contrary, feeding and oviposition behaviour of the willow leaf beetle *Plagiodera versicolora* Laicharting (Coleoptera: Chrysomelidae) was impaired by the rust fungus *Melampsora allii-fragilis* Kleb. infection (Simon and Hilker 2005). The negative effect on insect performance in plants infected with pathogens can also be caused by mycotoxins produced and released by fungi (Guo et al. 2014; Rostás and Hilker 2002).

Phytopathogens can also modify the plant responses by interfering with the activation of defence signalling pathways mediated by hormones, such as ethylene (ET), salicylic acid (SA) and jasmonic acid (JA). These pathways/molecules are involved in the resistance against both fungal pathogens and insects, although not all of them are elicited or effective against all the attackers (Fernandez-Conradi et al. 2018). Thus, it is known that infection with biotrophic pathogens induces the SA-dependent pathway, effective mainly against the same biotrophes as well as against sucking insects, while infection with necrotrophic pathogens activates the defences mediated by ET/JA, effective mainly against the same necrotrophes as well as against chewing insects (Fernandez-Conradi et al. 2018; Lazebnik et al. 2014; Pieterse et al. 2012). The induction of phytohormones by phytopathogens is associated with transcriptional reprogramming of defence genes, who enter in state of activation, ready to be transcribed after the interaction with an additional stress factor (De Vos et al. 2005; Espinas et al. 2016): this is the “priming” effect, that could affect insect behaviour and performance on plant previously infected by fungi.

Changes in pathogens-induced hormonal profile may influence the production of organic volatile compounds (VOCs) (Quaglia et al. 2012) and interfere in interactions between plants and herbivores. The emission of VOCs modified blends alters the attractiveness of plants for insects (Mann et al. 2012; Piesik et al. 2011; Sun et al. 2016) or has an effect on oviposition behaviour by female insects (Dötterl et al. 2009; Tasin et al. 2012). JA-induced volatiles are among the most important signals for plant indirect defence against insect herbivores, acting by attraction of their natural enemies; in addition, the JA-mediated pathway activation substantially affects the preference and performance of herbivores on plants (War et al. 2011). SA-induced VOCs are less studied in the host–herbivore interactions, where the volatile methyl salicylate (MeSA) has been shown to be especially associated with larval performance and oviposition behaviour (Groux et al. 2014; Ulland et al. 2008). Plants under simultaneous attack of pathogens and herbivores show a reduction or an increase in VOC production depending on whether the signalling pathway is SA- or JA-mediated (Cardoza et al. 2002; Rostás et al. 2006). Also, on plants subjected to double stress, plant volatiles induced by pathogens affects the recruitment of parasitoids (Shiojiri et al. 2001). In addition, it has been reported that pathogenic and non-pathogenic microorganisms produce VOCs themselves that impact on plant development and insect resistance (Moisan et al. 2019). These authors suggest that the VOCs emitted by pathogens represent a “warning” signal of the presence of a potential enemy and trigger the response of plants, while those produced by non-pathogenic microorganisms are recognized and used to predispose the plants to the contact of a potential mutualist (Moisan et al. 2019).

All these data clearly show that infections caused by fungal pathogens can alter the response of plants to insect infestation. Since the effects appear sometimes contradictory, depth studies on these tripartite interactions plant-pathogen-insect are of great importance also for developing new approaches to control herbivorous insects in nature.

In this work we studied the effect of pre-inoculation with fungal pathogens with different lifestyles, the necrotrophic *Botrytis cinerea* Pers. ex Fr. or the biotrophic *Golovinomyces orontii* (Castagne) V.P. Heluta, on *Arabidopsis thaliana* L. (Heynh) (ecotype Columbia-0; Col-0) plant defence against the insect herbivore *Eurydema oleracea* L. (Hemiptera: Pentatomidae). Both fungi produce disease and important yield losses in a wide variety of plant species (Dean et al. 2012). In the model plant *A. thaliana*, under controlled laboratory conditions, they cause infections that begin with conidium germination and, after penetration and colonization, end with sporulation, that represent the inoculum for new infection. During colonization, *B. cinerea* kills host cell by enzymes and toxins and feeds on dead plant tissues (Jiang et al. 2016), while *G. orontii* feeds on living host cell by a specialized cell called haustorium (Micali et al. 2011). Both the infection cycles are completed approximately in 4–5 days (Jiang et al. 2016; Micali et al. 2011). *E. oleracea* is an herbivore insect with piercing sucking mouthparts that belongs to Pentatomidae. This species is particularly harmful to Brassicaceae (Bohinc and Trdan 2012), and capable to cause significant injuries in *A. thaliana*, as demonstrated in our recent study (Ederli et al. 2020). In particular, we here examined if *B. cinerea* and/or *G. orontii* pre-inoculation affect *A. thaliana* susceptibility to *E. oleracea* in terms of feeding behaviour. Moreover, to better clarify the effect of pathogenic fungi on *E. oleracea*, we carried out a comparative study on plant defence responses to pathogen inoculation and/or insect infestation by analysing the expression of marker genes for SA- and JA-dependent signalling pathways. Finally, we evaluated the impact of VOCs emitted by pathogen-inoculated plant on host plant choice and feeding preference of insect.

## Materials and methods

### Plants growth

Columbia (Col-0) is one of the most popular Arabidopsis accessions; its genome has been entirely sequenced (The Arabidopsis Genome Initiative 2000) and a collection of T-DNA insertion mutants is available for this accession, making it particularly suitable as a molecular model for genetic studies. Morphological and physiological traits of Col-0 ecotype are described in Passardi et al. (2007). Sterilized seeds of the ecotype Col-0 were vernalized for 2 days at 4 °C and sown in plastic pots (5.5 cm diameter) in sterile soil mix (Patzer Einheitserde, Manna Italia, Bolzano, Italy). Plants were grown in a climatic chamber at 60–75% relative air humidity and 24 °C/19 °C temperature for 12 h day/12 h night photoperiod. During day, the lights were set to an intensity of 200 µmol m^− 2^ s^− 1^. Plants were watered by sub-irrigation once a week to avoid water stress.

### Plant pathogens and insect

Necrotrophic pathogen *B. cinerea* isolate as reported by Ederli et al. (2015) and biotrophic pathogen *G. orontii* isolate (kindly supplied by Prof. Aleš Lebeda and Dr. Božena Sedláková, Department of Botany, Palacký University of Olomouc - Czech Republic) as reported by Bianchet et al. (2019) were used.

A colony of *E. oleracea* obtained from adults collected in the field close to Perugia (Italy), was maintained in a growth chamber into isolation cages (30 × 30 cm). Insects were reared at 24 ± 2 °C temperature, 70% relative humidity and 16/8 h light/dark. The colony was established for 2 months prior to the onset of experiments to ensure that insects were suitably adapted to new conditions. Both leaves and inflorescences of different cruciferous plants were used for insects feeding. In all experiments unmated female insects were used.

### Plants inoculation and/or infestation

Experiments were carried out on 3-weeks-old *A. thaliana* Col-0 plants.

For *B. cinerea*, inoculum preparation and inoculation were performed according to Ederli et al. (2015). In particular, inoculation was performed deposing two 5-µL drops of spore suspension (5 × 10^5^ conidia mL^− 1^) onto single leaf.

For inoculation with *G. orontii*, conidia harvested from infected *Cucurbita* spp. used as inoculum-maintaining plants, were suspended in sterilized deionized water added with 0.04 % (v/v) Tween 20^®^ (Sigma-Aldrich Inc., St. Louis, USA) to a final concentration 5 × 10^5^ conidia mL^− 1^, as measured by hemocytometer; the conidial suspension was sprayed on the surface of a single leaf using a hand atomizer until run-off (Bianchet et al. 2019).

All the inoculated plants were placed into the growth chamber, at the conditions above reported, except being kept under 100% relative air humidity for the first 24 h post-inoculation.

*E. oleracea* infestation was carried out after the insect was left without food for 24 h. One unmated insect female was placed on a single leaf within a clip-cage, obtained from a plastic Petri dish (3.8 cm diameter; 1 cm high) with a mesh-covered hole (3 cm diameter) and with the rim covered by a sponge ring to prevent damage to the leaf. After 24 h of feeding, insect was removed.

For the tripartite interactions, following the methods above reported, plants inoculated with *G. orontii* or *B. cinerea* were infested by *E. oleracea* at 1 day post-inoculation (dpi). At this time (1 dpi), Arabidopsis plants inoculated with *B. cinerea* showed very slight leaf symptoms such as chlorotic spots 0.5–1 mm in diameter, whereas those infected by *G. orontii* were free from disease symptoms. Batches of leaves collected from plants sprayed only with sterile deionized water added with 0.04% (v/v) Tween 20 and treated with empty clip-cage kept on the leaf for 24 h were used as control.

### Insect feeding behaviour

In order to study the effect of the infection with the biotrophic or the necrotrophic pathogen on plant response to the following *E. oleracea* infestation, the insect feeding behaviour was assessed on plant infested with *E. oleracea* at 1 dpi with *G. orontii* or *B. cinerea*. In particular, leaf herbivore damage was quantified 1 day after exposure to *E. oleracea*, immediately after insect removal (corresponding to 2 dpi), in both uninfected (control) and infected plants, by observation with a stereomicroscope (WILD M420, Switzerland) and analysis by the software ImageJ (Schneider et al. 2012). This method allowed to measure the surface of the damaged portion of the leaf, represented by whitish area localized around the points where the insect inserted its stylets and injected the saliva.

### Gene expression analysis by RT-qPCR

In order to assess the effect of inoculation and/or infestation on expression of SA-responsive pathogenesis-related protein 1a (*PR1a*) and JA-responsive plant defensin 1.2 (*PDF1.2*) genes, leaf samples were taken from: (a) healthy plants, uninoculated and uninfested, used as control, (b) plants inoculated with *G. orontii* or *B. cinerea*, (c) plants infested with *E. oleracea*, (d) plants inoculated with *G. orontii* or *B. cinerea* and infested with *E. oleracea*. In particular, sampling was carried out at 1, 2 and 3 dpi on plants inoculated with pathogens, at 1 and 2 days post-infestation on plants infested with herbivore (immediately after- and 1 day after- insect removal, corresponding to 2 and 3 dpi, respectively), at all these time in plants inoculated with each pathogen and infested with herbivore, at any time in the healthy control plants. Samples were immediately frozen in liquid nitrogen. Total RNA was extracted and isolated from frozen leaf tissue (100 mg) using PureLink™ RNA Mini Kit (Thermo Scientific, Waltham, USA), according to the manufacturer instructions. The RNA concentration was checked using the Qubit RNA BR (Broad-Range) assay kit with the Qbit™ 3.0 Fluorometer (Thermo Fisher Scientific). First-strand cDNA was synthesized from 1 to 2 µg of total RNA using PrimeScript™ RT-PCR Kit (Takara, Shiga, Japan), according to the manufacturer’s protocol. The resulting cDNA was diluted fivefold for quantitative real-time PCR (RT-qPCR) analyses that were carried out with 8.0 ng cDNA in a final volume of 20 µL using the SYBR Premix Ex Taq II reagent (Takara, Shiga, Japan), according to the instruction manual. The RT-qPCR was performed using the CFX96 detection system (Bio-Rad, Hercules, CA, USA). The thermal cycling conditions are as follows: for *PR1a* gene initial denaturation at 95 °C for 3 min, followed by 40 repeated cycles of 95 °C for 20 s, 60 °C for 20 s, and 72 °C for 15 s, while for *PDF1.2* gene initial denaturation at 95 °C for 3 min, followed by 40 repeated cycles of 95 °C for 20 s, 58 °C for 20 s, and 72 °C for 20 s. A melting curve analysis was carried out from 55 to 95 °C in 0.5 °C steps and 10 s dwell time to confirm that the fluorescence resulted from a single amplicon and did not represent primer dimer or nonspecific products. The primers used were: *PR1a* gene (At2g14610) forward 5′-CGAAAGCTCAAGATAGCCCAC-3′ and reverse 5′-AAACTCCATTGCACGTGTTCG-3′ primers and *PDF1.2* gene (At5g44420) forward 5′-TTTGCTGCTTTCGACGCAC-3′ and reverse 5′-TAACATGGGACGTAACAGATA-3′ primers. Relative amounts of the transcripts were calculated by the 2^− ΔΔCt^ method (Livak and Schmittgen 2001), using the Arabidopsis Elongation factor-1α (At1g07940) gene (forward primer 5′-AGTGGTCGTACAACCGGTATTGT-3′ and reverse primer 5′-TGGTGGTCTCGAACTTCCAG-3′) as internal standard.

### Effect of treatment with volatile organic compounds emitted by *B. cinerea* infected plants on *E. oleracea* feeding preference

Finally, the impact of VOCs emitted by *B. cinerea-*inoculated plants on leaf herbivore damage was evaluated by a dual choice experiment using a system well described in Piersanti et al. (2020). Briefly, volatile compounds emitted from Arabidopsis plants at 1 dpi with *B. cinerea* were collected. A couple of Arabidopsis leaves belonging to two different plants was placed inside a clip-cage, as previously described for the insect infestation. The abaxial surface of each leaf was in contact with the filter paper strip (15 mm × 15 mm, Whatman No. 1) used as odour cartridge. The filter paper associated to one leaf was impregnated with 20 µL of VOCs extract collected from *B. cinerea* inoculated plants, while the filter paper associated to the other leaf was impregnated with 20 µL of hexane, the solvent used for eluting VOCs. Feeding preference was assessed as leaf area damaged, quantified as described for insect feeding behaviour.

### Statistics

The leaf injury values related to the effect of *G. orontii* or *B. cinerea* infection on *E. oleracea* feeding behaviour and on treatment with VOCs emitted by *B. cinerea* inoculated plants on insect preference were tested for statistical difference considering 22–25 and 18 replicates, respectively, using Student’s *t* test for dependent samples.

Instead, data related to the effect of pathogen inoculation and/or insect infestation on *PR1a* and *PDF1.2* gene expression were submitted to one-way analysis of variance (ANOVA), followed by Tukey’s test. Experimental designs are shown in the figure legends.

## Results

### Effect of pathogens inoculation on *E. oleracea* feeding behaviour

**Fig. 1:**
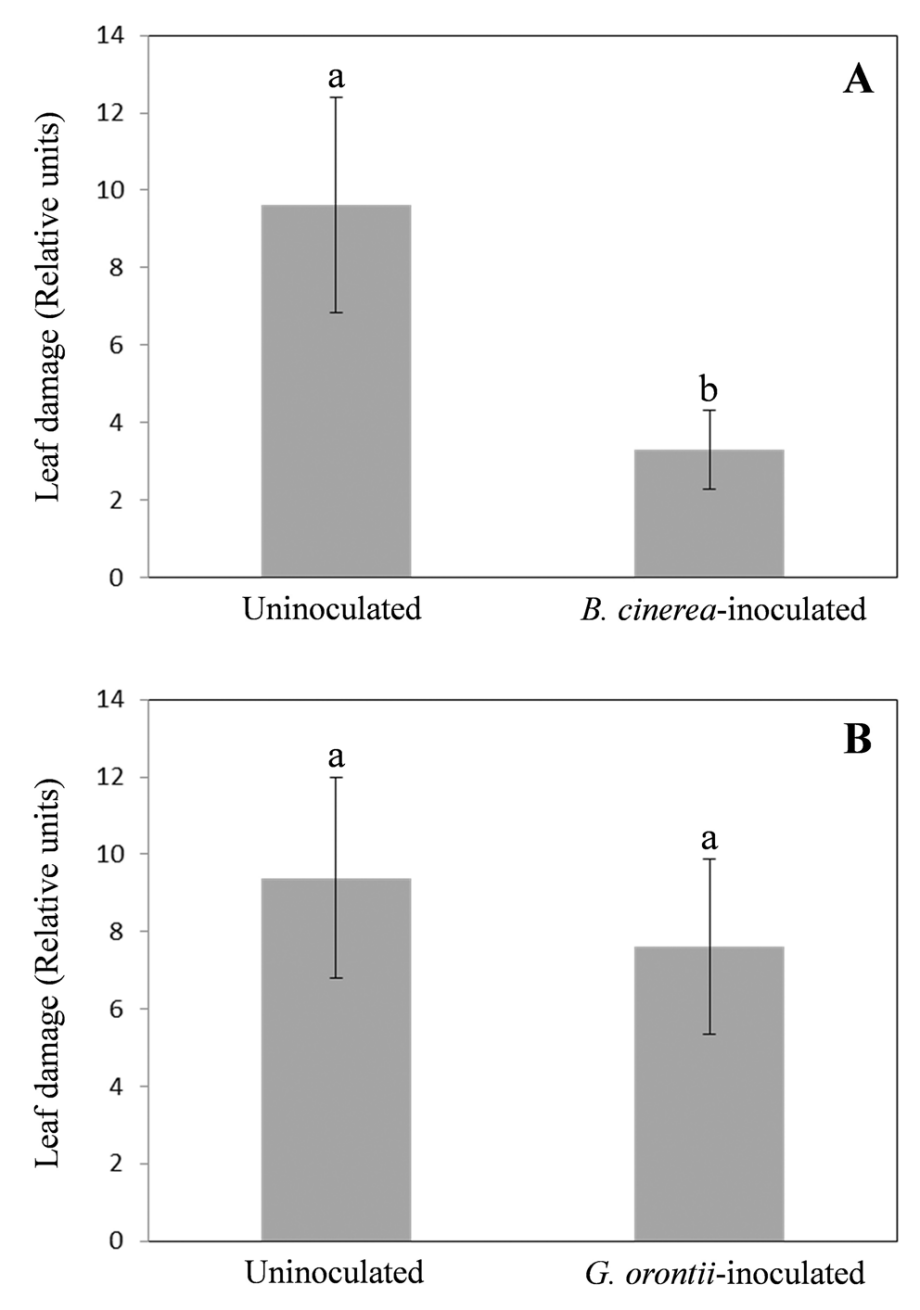
Effect of pathogens inoculation on leaf damage caused by *E. oleracea* feeding. Arabidopsis leaf injuries were assessed at 1 day after exposure to one unmated female of *E. oleracea* in both uninoculated plants (control) and plants inoculated 1 day before with *B. cinerea* (**a**) or *G. orontii* (**b**). The data were the mean ± SE of 22–25 replicates. Different letters indicate statistically different mean values (p ≤ 0.001; Student’s t test) for dependent samples

Fungal pathogens with different lifestyles showed a different impact on the susceptibility of *A. thaliana* Col-0 plants to the insect *E. oleracea*. Indeed, on plants inoculated with the necrotrophic *B. cinerea* the herbivore feeding behaviour was altered being leaf damage significantly reduced with respect to those on uninoculated, control plants (Fig. 1a). In particular, the reduction in leaf injury was over than 65% to that of the control plants (Fig. 1a). On the contrary, on Arabidopsis plants inoculated with the biotrophic fungus *G. orontii* no differences in the herbivore feeding choice were recorded: in this case, the foliar damage was only slightly, but not significantly, reduced in the inoculated plants with respect to those uninoculated (Fig. 1b).

### Analysis of SA- and JA-responsive defence genes after pathogen inoculation and/or insect infestation

Since phytopathogens can interfere with the hormonal signalling pathways typically involved in plant defence and alter their response to subsequent stresses, including insects, we here studied the expression of the SA-dependent *PR1a* and JA-dependent *PDF1.2* genes in Arabidopsis plants after pathogens inoculation and insect infestation, alone or combined. Indeed, in several studies dealing with the induced resistance these two genes are commonly used as marker of SA- and JA- signalling pathways in *A. thaliana*, respectively (Ederli et al. 2020; Ellis et al. 2002; Leon-Reyes et al. 2010). Moreover, among the *PDF* genes, *PDF1.2* has been chosen since its expression has been reported at The Arabidopsis Information Resource (TAIR) (http://www.arabidopsis.org) to be involved in *A. thaliana* leaves response to insect and necrotrophic pathogen *B. cinerea* but not to the biotrophic pathogen *G. orontii* (syn. *Erysiphe orontii*), thus it was particularly suitable in this study.

**Fig. 2:**
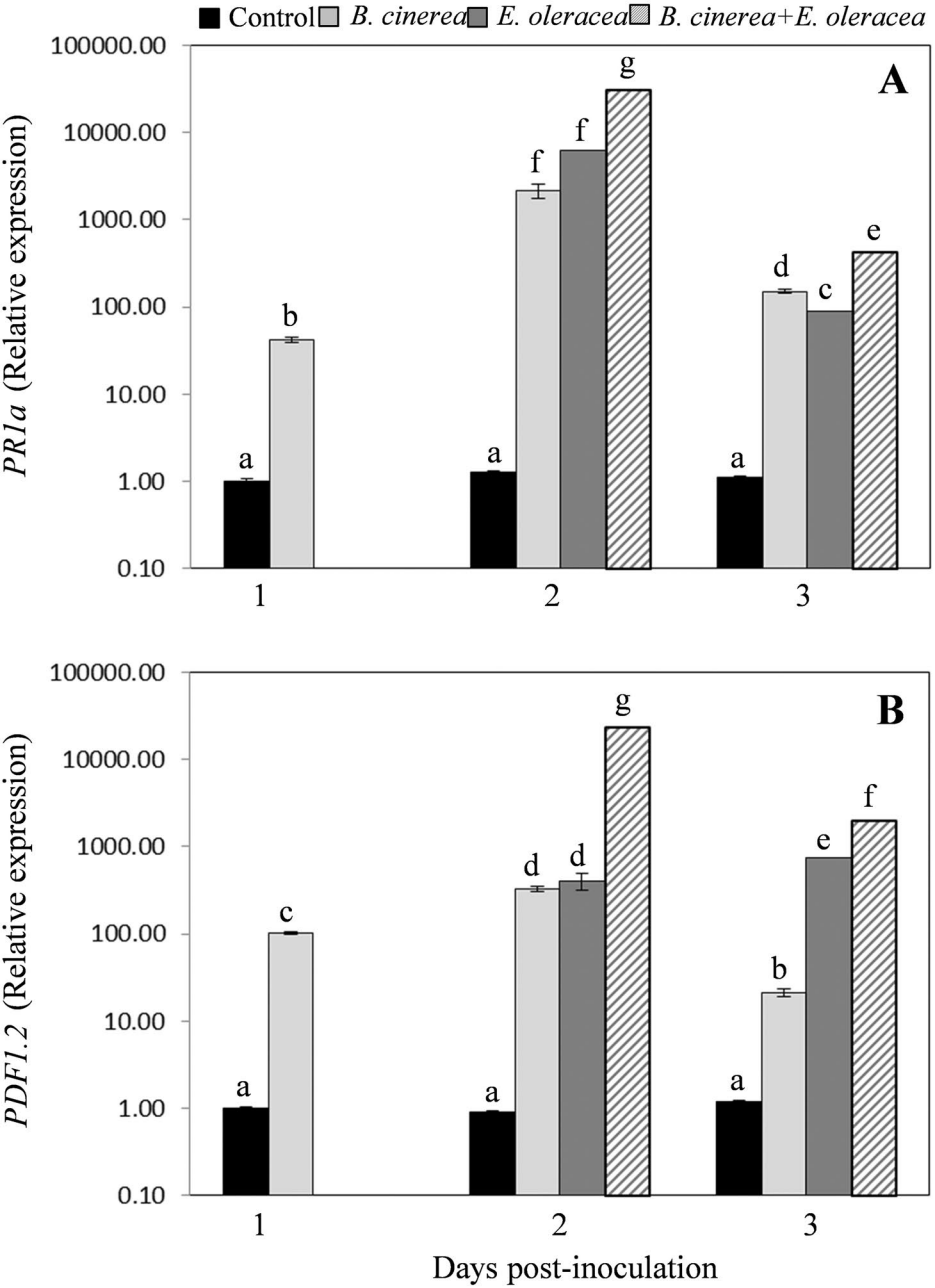
Quantitative real-time PCR analysis of SA- and JA-responsive genes after *B. cinerea* inoculation and *E. oleracea* infestation, alone or combined. Relative expression levels of SA-responsive *PR1a* gene (**a**) and JA-responsive *PDF1.2* gene (**b**) measured on healthy plants (control) and on plants: inoculated with *B. cinerea* at 1, 2 and 3 days post inoculation (dpi), exposed to *E. oleracea* at 1 day after herbivore infestation (corresponding to 2 dpi) and 1 day after insect removal (corresponding to 3 dpi), exposed to *E. oleracea* at 1 dpi with *B. cinerea*, at 1 day after herbivore infestation (corresponding to 2 dpi) and 1 day after insect removal (corresponding to 3 dpi). All data reported are the mean ± SE of three independent experiments. In each experiment three biological replicates per treatments, each obtained from six individual plants, and three technical replicates were analysed. Different letters indicate statistically different mean values (p ≤ 0.001; ANOVA one-way, Tukey’s HSD tests)

Considering *B. cinerea*, with respect to the healthy (uninoculated/uninfested) control plants, on inoculated plants the *PR1a* transcript levels already significantly increased at 1 dpi, peaked at 2 dpi and then decreased at 3 dpi, however remaining very high (more than 100 times the controls) (Fig. 2a). On *E. oleracea* infested plants, *PR1a* transcript level was strongly up-regulated with respect to control both at 1 day post-infestation (corresponding to 2 dpi) and at 1 day after insect removal (corresponding to 3 dpi), although at 1 day after insect removal the *PR1a* transcript level drastically declined (Fig. 2a). Finally, plants both inoculated with *B. cinerea* and infested with *E. oleracea* showed a *PR1a* induction profile similar to those reported for plants just *B. cinerea*-inoculated or *E. oleracea*-infested. However, in the tripartite combination plant-pathogen-insect a significantly higher level of *PR1a* transcripts was observed, in comparison with *B. cinerea*-inoculated or *E. oleracea*-infested plants (Fig. 2a), indicating a synergistic effect of the two biotic stresses on the induction of SA-mediated signalling pathway.

The JA-responsive gene *PDF1.2* transcription was also stimulated by *B. cinerea* inoculation and/or *E. oleracea* infestation (Fig. 2b), following the trend above described for *PR1a* gene transcripts.

**Fig. 3:**
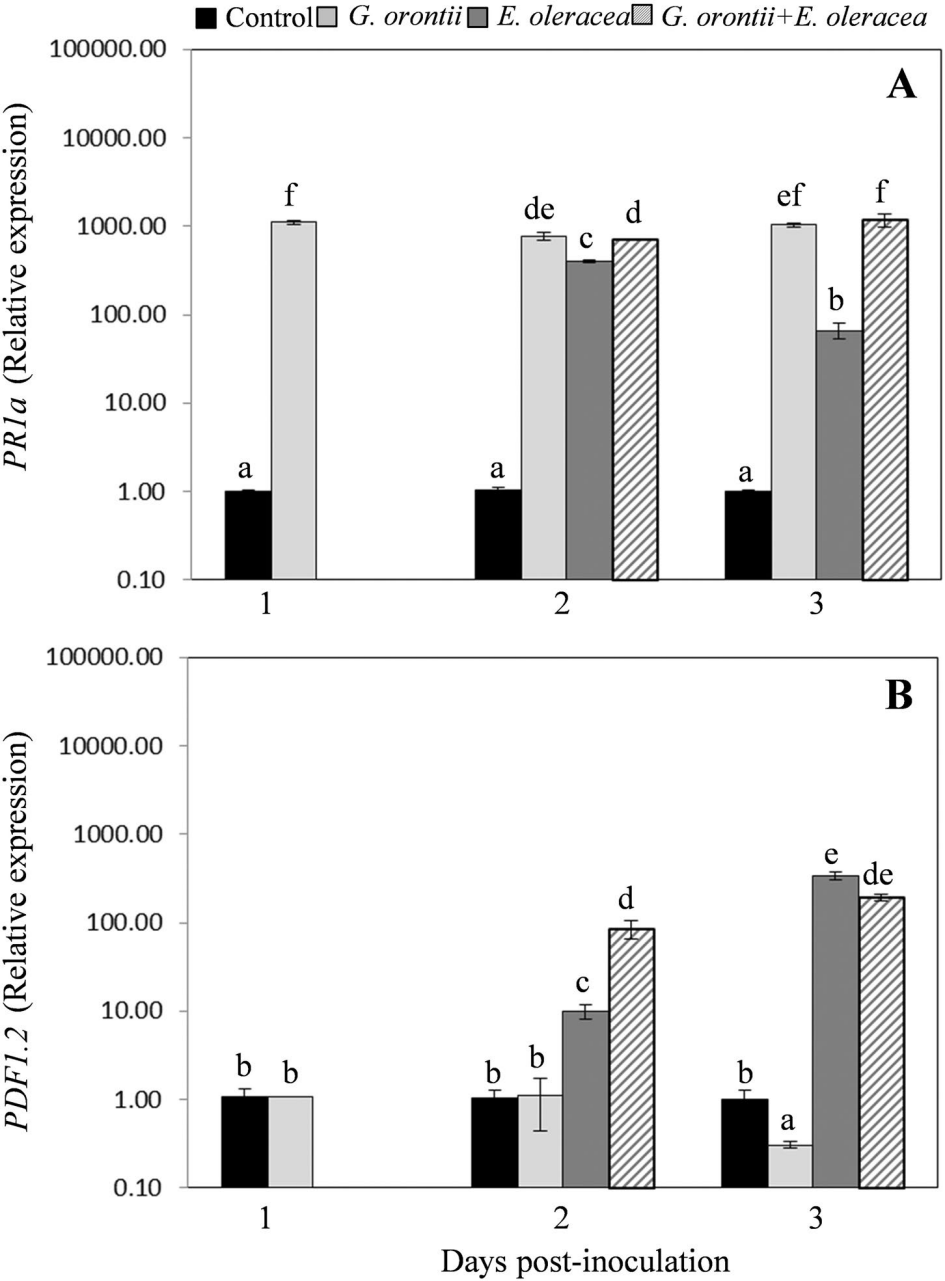
Quantitative real-time PCR analysis of SA- and JA-responsive genes after *G. orontii* inoculation and *E. oleracea* infestation, alone or combined. Relative expression levels of SA-responsive *PR1a* gene (**a**) and JA-responsive *PDF1.2* gene (**b**) were measured on healthy plants (control) and on plants: inoculated with *G. orontii* at 1, 2 and 3 days post inoculation (dpi), exposed to *E. oleracea* at 1 day after herbivore infestation (corresponding to 2 dpi) and 1 day after insect removal (corresponding to 3 dpi), exposed to *E. oleracea* at 1 dpi with *G. orontii* at 1 day after herbivore infestation (corresponding to 2 dpi) and 1 day after insect removal (corresponding to 3 dpi). All data reported are the mean ± SE of three independent experiments. In each experiment, three biological replicates per treatments, obtained from six individual plants, and three technical replicates were analysed. Different letters indicate statistically different mean values (p ≤ 0.001; ANOVA one-way, Tukey’s HSD tests)

As expected, *G. orontii* inoculation strongly triggered *PR1a* transcripts accumulation (Fig. 3a) while did not affected the *PDF1.2* transcript levels, except for a reduction at 3 dpi (Fig. 3b). In this set of experiments the transcriptional profile of both genes above reported after *E. oleracea* infestation was also confirmed (Fig. 3). Moreover, in Arabidopsis plants simultaneously exposed to pathogen and insect (*G. orontii* + *E. oleracea*) a significant increase in transcripts of both *PR1a* and *PDF1.2* genes was evidenced at all times examined (1 day post-infestation and 1 day after insect removal, corresponding to 2 and 3 dpi, respectively; Fig. 3).

### Impact of treatment with *B. cinerea*-induced VOCs on *E. oleracea* preference

Since only *B. cinerea-* and not *G. orontii*-inoculation significantly affect the susceptibility of *A. thaliana* to *E. oleracea* and induced the JA-mediated pathway, notoriously involved in the *de novo* synthesis of plant VOCs, so much so that the ability to biosynthesize these compounds appears massively reduced in plants impaired in the JA biosynthetic pathway (Degenhardt et al. 2010; Quaglia et al. 2012; van Schie et al. 2007), we here tested the effect of treatment with VOCs collected from Arabidopsis plants at 1 dpi with *B. cinerea* on herbivore preference.

**Fig. 4:**
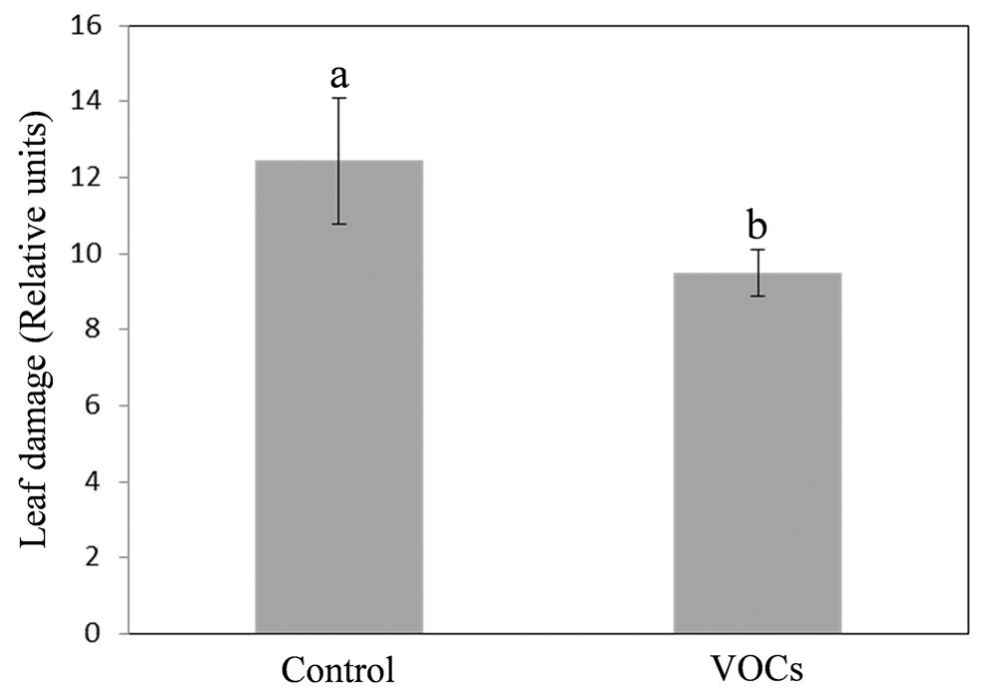
Effect of VOCs emitted by *B. cinerea* infected plants on *E. oleracea* preference. A couple of Arabidopsis leaves belonging to two different plants was placed inside a clip-cage and the abaxial surface of each leaf was put in contact with the moist filter paper strip impregnated with 20 µL of VOC extract collected from *B. cinerea* inoculated plants, or dipped in hexane solution (control). Leaf damage was quantified 24 h after infestation by one unmated insect female of *E. oleracea* inside each clip-cage using image analysis software ImageJ. The data were the means ± SE. Different letters indicate statistically different mean values (p ≤ 0.001; Student’s t test) for dependent samples

Effectively, pre-treatment with VOCs conferred protection against subsequent herbivore infestation: on the pre-treated leaves the incidence of the *E. oleracea* attack was lower in comparison with those on the untreated controls (only 72% versus 100%). Moreover, when the insect fed on VOCs pre-treated plants the leaf damage area was significantly lower in comparison with controls (Fig. 4).

## Discussion

In nature, plants have to deal with multiple biotic stresses and their defence is the result of complex interactions between unique and common responses to individual stress or stress combination. All leads to physiological and molecular adaptation strategies which are similar or very different, or even contrasting, from those adopted under individual stresses. Understanding these multipartite interactions is essential to develop on one side varieties with broad spectrum stress tolerance and on the other side combined pest management strategies.

In recent years, plant–microbe–insect interactions have been the subject of various studies, many of which concerned the role of beneficial microorganisms in plant immunity to insect species. Thus, it has been found that arbuscular mycorrhizal fungi impact on plant nutrient dynamics, secondary metabolism and defence traits, influencing insect behaviour (Frew and Price 2019), and the colonization of roots by rhizobia can induce resistance in soybean to aphids (Dean et al. 2009). Beneficial microbes can also elicit induced systemic resistance (ISR) that primes plant for enhanced defence against future attack by a broad range of herbivorous insects (Partida-Martinez and Heil 2011; Pieterse et al. 2014; Pineda et al. 2013).

Additionally, several studies have dealing with the effect of pathogenic microorganisms on plant response to insects. These studies shown conflicting results, with both positive (Eberl et al. 2020) or negative (Desurmont et al. 2016; Ngah et al. 2018; Yang et al. 2013) impact of fungal infection on insect behaviour. To date, many aspects of these interactions have not been fully elucidated and require further investigations.

For this reason, we here investigated the effect of pre-inoculation with two fungal pathogens different in lifestyle, the necrotroph *B. cinerea* and the biotroph *G. orontii*, on the herbivore *E. oleracea* behaviour. All of them produce enormous economic damage on crucifers worldwide (Bohinc and Trdan 2012; Jiang et al. 2016).

A marked difference in the plant response to the insect infestation was evidenced, since leaf damage caused by *E. oleracea* was significantly reduced by *B. cinerea* but not *G. orontii* pre-inoculation.

To better investigate the different effect of the two pathogens on Arabidopsis susceptibility to *E. oleracea*, we then studied the expression of the *PR1a* and *PDF1.2* genes as marker of SA-dependent and JA-dependent defence pathways. Notoriously, during the infective process the two pathogens require different hormonal pathways as key regulators of plant immunity (Fernandez-Conradi et al. 2018; Glazebrook 2005). While the activation of the SA-dependent pathway is essential against biotrophic pathogens and sucking insects, the defence against necrotrophic pathogens and chewing insect pass mainly through the JA-signalling activation (Fernandez-Conradi et al. 2018; Glazebrook 2005), although the role of the JA pathway against biotrophic pathogens as well as those of SA pathway against necrotrophs have also been demonstrated (Ellis et al. 2002).

Our data showed that while *G. orontii* only activated the SA-related pathway, *B. cinerea* or *E. oleracea* stimulated both the SA- and JA-dependent signalling pathways, thus indicating the involvement of the two hormonal way in Arabidopsis defence response to the last two stresses. The role of SA-pathway in *Golovinomyces* spp.- and *B. cinerea-*inoculated Arabidopsis plant, as well as the role of SA- and JA-pathways in *E. oleracea* infested plants, fit with those previously reported by authors (Ederli et al. 2020), while the role of JA/ET pathway on *B. cinerea* inoculated Arabidopsis plants was previously shown by Moffat et al. (2012). According to Ederli et al. (2020), our data also confirmed that insect removal resulted in a reduction in the transcript level of *PR1a* and in an increase in the *PDF1.2* gene transcripts.

Furthermore, here we shown for the first time that combined exposure to pathogen and herbivore amplified the induction of the two genes transcripts, indicating a synergistic action of the two biotic stresses in the induction of the defence pathways. In particular, this was true in *B. cinerea–E. oleracea* interaction for both *PR1a* and *PDF1*.2 genes at 1 day post-infestation (2 dpi) and at 1 day after insect removal (3 dpi), and for *G. orontii–E. oleracea* interaction for *PDF1.2* gene at 1 day post-infestation (2 dpi).

We previously highlighted, also using different mutant lines, that the defence of Arabidopsis plants against *E. oleracea* was associated with the JA-dependent pathway, while SA facilitated the herbivore’s fitness (Costarelli et al. 2020; Ederli et al. 2020). Therefore, the reduced susceptibility of the *B. cinerea*-inoculated Arabidopsis plants to insect could be due to the activation of the JA-related pathway, lacking in *G. orontii*-inoculated plants. Basit et al. (2019) recently showed that the necrotrophic fungus *B. cinerea* produced an elicitor (PeBC1) that induces resistance to herbivores in beans. In Arabidopsis Col-0 plants, exogenous treatment with this elicitor did not induce *PR1* but strongly stimulated the expression of *PDF1.2*; the inductive effect of treatment on plant response persisted for a long time (Zhang et al. 2014). In addition, treatment increased the resistance to *B. cinerea* also in *npr1* and *NahG* mutant lines with inhibited SA-dependent pathway, but not in *ein2* and *coi1* mutant lines with inhibited JA/ET-dependent pathway, indicating that the elicitor acted through this last pathway. In our experiments the JA-mediated pathway induced by *B. cinerea*, also in synergy with *E. oleracea* feeding according to the priming effect of the *B. cinerea* inoculation (De Vos et al. 2005; Espinas et al. 2016), not only remained active after the herbivore removal, but even increased, suggesting that the signalling persisted for a long time, probably providing significant long-term resistance to further attacks by herbivores. In support of this last hypothesis, very preliminary experiments showed that Arabidopsis plants infested with *E. oleracea* developed lower susceptibility to a second attack by the insect (data not shown).

Although JA plays a critical role in Arabidopsis protection against *E. oleracea* how the activation of JA-signalling led to an increased resistance had not been defined. To elucidate a possible mode of action, in this work we focused on the role of JA pathway as regulator of plant volatile emission (Ament et al. 2004; Jiang et al. 2017; Tanaka et al. 2014). Indeed, previous research works demonstrated that plant VOC production can be strongly affected by the presence of microorganisms, altering the performance of insect attackers, and that the protection afforded by VOCs can be highly effective, as these compounds can remove herbivores from the plant or disable them at an early stage of the attack, significantly limiting the damage (Desurmont et al. 2016; Mauck et al. 2015; Sun et al. 2016). Consistent with the literature, in our investigations, treatment with VOCs emitted by plants inoculated with the necrotrophic fungus *B. cinerea* modified the plant responses to the following infestation with *E. oleracea*, both inhibiting herbivore host plant choice and feeding injury.

In conclusion, in this study we reported experimental evidences of how an early stage infection with a necrotrophic pathogen can alter subsequent plant interactions with insects. Specifically, *B. cinerea* inoculation reduced the susceptibility of Arabidopsis to the herbivore *E. oleracea* quickly triggering the JA-dependent pathway which, in turn, induced the production of VOCs, key factors in plant defences. The signalling persisted for a long time, making plants less susceptible to further challenges. These results give new insights into the understanding of plant responses to the various biotic components of the ecosystem and their complex interactions, also providing knowledge to develop tools to improve field productions.
